# Burnout Syndrome in Paediatric Oncology Nurses: A Systematic Review and Meta-Analysis

**DOI:** 10.3390/healthcare8030309

**Published:** 2020-08-29

**Authors:** Emilia Inmaculada De la Fuente-Solana, Laura Pradas-Hernández, Alicia Ramiro-Salmerón, Nora Suleiman-Martos, José Luis Gómez-Urquiza, Luis Albendín-García, Guillermo Arturo Cañadas-De la Fuente

**Affiliations:** 1 Brain, Mind and Behavior Research Center (CIMCYC), University of Granada, Campus Universitario de Cartuja s/n, 18071 Granada, Spain; edfuente@ugr.es; 2 San Cecilio Clinical University Hospital, Andalusian Health Service, Avenida de la Investigación s/n, 18016 Granada, Spain; 3 Virgen de las Nieves University Hospital, Andalusian Health Service, Avenida de las Fuerzas Armadas, nº6, 18014 Granada, Spain; aligrana_@hotmail.com; 4 Department of Nursing, Faculty of Health Sciences, University of Granada, Calle Cortadura del Valle s/n, 51001 Ceuta, Spain; norasm@ugr.es; 5 Department of Nursing, Faculty of Health Sciences, University of Granada, Avenida de la Ilustración s/n, 18016 Granada, Spain; jlgurquiza@ugr.es (J.L.G.-U.); gacf@ugr.es (G.A.C.-D.l.F.); 6 La Chana Health Center, Granada Metropolitan District, Andalusian Health Service, Calle Joaquina Eguaras, nº 2, Edificio 2 1ª planta, 18013 Granada, Spain; lualbgar1979@ugr.es

**Keywords:** burnout, nursing, paediatric oncology, prevention, risk factors

## Abstract

*Purpose*: To determine levels of burnout among paediatric oncology nurses, and the risk factors that may influence its development. *Method*: A literature review with meta-analysis was conducted, via a search in the PubMed, CINHAL, Scopus, ProQuest (Health and Medical Complete), Scielo and PsycINFO databases, using the search equation: “Nurs* AND burnout AND oncology AND pediatric”. *Results*: The final sample of selected studies was of eight articles. All were quantitative studies of paediatric oncology nurses, using the Maslach Burnout Inventory, written in English or Spanish. No search restrictions were established on the year of publication. The eight studies reported moderate–high levels of burnout in each of its three dimensions. These dimensions were all related to the characteristics of the profession, in terms of complexity, shifts and workload, and to sociodemographic variables such as marital status, work experience, age and gender. The prevalence meta-analytical estimation for a sample of *n* = 361 nurses were 37% for high emotional exhaustion, 16% for high depersonalisation and 27% for low personal fulfilment. *Conclusions*: Most paediatric oncology nurses present moderate–high levels of burnout. Therefore, strategies and interventions should be identified and implemented to protect these workers from the syndrome.

## 1. Introduction

Burnout syndrome is a condition that arises from continuous occupational stressors which provokes negative effects, both on the individuals affected and also on their environment [1]. According to Maslach and Jackson (1981) [2], it consists of three dimensions: high emotional exhaustion (EE), high depersonalisation (D) and low personal accomplishment (PA), and mainly impacts persons whose work involves dealing with other people. Among healthcare workers, EE appears as a state of extreme fatigue, in which nurses (for example) become less sensitive to others and lose interest in them; D results in the development of negative behaviour towards patients, sometimes accompanied by cynicism and indifference; finally, low PA is the negative assessment of one’s own work, leading to reduced interest and impaired performance [3]. Burnout syndrome can be assessed by the Maslach burnout inventory (MBI), which was designed to measure the three dimensions of the syndrome [2].

Burnout is considered an occupational disease. It may also reflect inherent psychological problems, such as depression and anxiety, and sometimes those affected by it may be forced to leave their jobs [1]. Its effects have been observed among diverse occupational groups, including firefighters [4], police officers [5,6], military personnel [7], teachers [8,9] and, above all, healthcare workers [10,11,12,13].

Burnout has a strongly negative impact on healthcare workers, especially nurses, who are exposed to many personal and work-related risk factors that facilitate the development of the syndrome [14,15,16,17]. This vulnerability directly affects the level of care provided, reducing nurses’ productivity and diligence towards their patients [18]. This is due to the working environment of these professionals, as it has complex relationships and puts them in contact with death and with the pain and suffering of patients and their families. Conflicts often appear in this complex environment, both at the organisational level with colleagues or managers, and with patients and their families [19]. All these stressors at work are present in the day-to-day of health professionals, making nurses a risk group for burnout development [20]. The World Health Organization (WHO) defines stress as a ‘set of physiological reactions that prepares the body for action’. From the occupational point of view, this occupational stress arises due to an alteration between the professional, the work that they perform and the organisation where they work. In this way, the need to face the problem without sufficient resources arises, generating a stressful situation with consequent physical and psychological manifestations deriving from it. In fact, burnout is described by the WHO as a syndrome resulting from chronic stress in the workplace, which has not been successfully managed and is characterised by the three dimensions discussed previously [21].

The prevalence of the disorder among these professionals is high, showing that up to 25% of nursing professionals would be affected by it [22]. Other authors inform of higher percentages and affirm that these levels of affection can be higher [23]. It has been showed that there is a higher prevalence of affected nursing personnel in certain units, such as, emergency [24], critical care [25], primary healthcare [26], mental health [27], medical area [28] and oncology [29], among others.

In this context, nurses working in a hospital oncology unit may be more susceptible to burnout, due to facing specific risk factors such as direct contact with death and pain suffered by patients and their families, as well as excessive workloads, possible conflicts with other healthcare workers and a perceived lack of social support [29,30]. These issues may be further exacerbated in especially complex fields such as paediatric oncology, where the death of child patients is a trauma that must routinely be borne [31]. 

Therefore, if high levels of stress are maintained daily, the problem of burnout will be very present in paediatric oncology nurses. This situation will not only affect nurses, but will lead to problems in the workplace. The most frequent problems will be a decrease in job performance by affected nurses and personal well-being, leading to various kinds of complications such as a decrease in patient safety and a decrease in quality of care [23].

## 2. Study Aims

To determine levels of burnout among paediatric oncology nurses, and the risk factors that may influence its development. 

## 3. Material and Method 

### 3.1. Search Sources and Search Strategy

A systematic literature review with meta-analysis was conducted, following the recommendations of the PRISMA statement [32]. The following databases were used in this search: PubMed, CINHAL, Scopus, ProQuest (Health and Medical Complete), Scielo and PsycINFO. The search equation applied was “Nurs* AND burnout AND oncology AND pediatric”, using words from the Medical Subject Headings (DeCS in Spanish). The search was conducted in June 2020.

### 3.2. Inclusion and Exclusion Criteria

The criteria for the studies selection were the following: quantitative primary studies about burnout in paediatric oncology nurses, using the MBI for burnout assessment and publication in English or Spanish. No restriction was applied on the year of publication in order to find as many studies as possible. Qualitative studies, doctoral theses and quantitative studies with a mixed sample (i.e., in which separate data for the paediatric oncology nursing service were not provided) were excluded.

### 3.3. Study Selection and Procedure

The following process was conducted to select the studies: duplicates were eliminated, and appropriate studies were then chosen according to their title and abstract. The full text was then read, and finally a backward and forward search was conducted with the selected studies to increase the number of articles considered. The level of evidence and the degree of recommendation were assessed using the Oxford Centre for Evidence-Based Medicine classification (OCEBM) [33]. The search, selection and coding process was conducted independently by two researchers, and inter-observer reliability was checked by reference to the intraclass correlation coefficient and Cohen’s kappa coefficient. A third member of the team was consulted in the case of disagreement. 

### 3.4. Variables and Data Encoding

For each study, the following data were encoded for analysis: first-named author; year of publication; country of the study; study method, type of MBI (Human Services Survey vs. General Survey); sample size of paediatric oncology nurses; number of nurses presenting high EE, high D and low PA; and burnout risk factors presented in the study.

### 3.5. Data Analysis

The meta-analysis was conducted using StatsDirect software (version 3, StatsDirect Ltd., Cambridge, UK), following a sensitivity analysis and the evaluation of possible publication bias according to Egger’s linear regression. The meta-analytic estimate of prevalence was obtained from three meta-analyses of random effects, one for each dimension of burnout. Heterogeneity was determined according to the Chocran Q values and the I^2^ index.

## 4. Results

**Figure 1:**
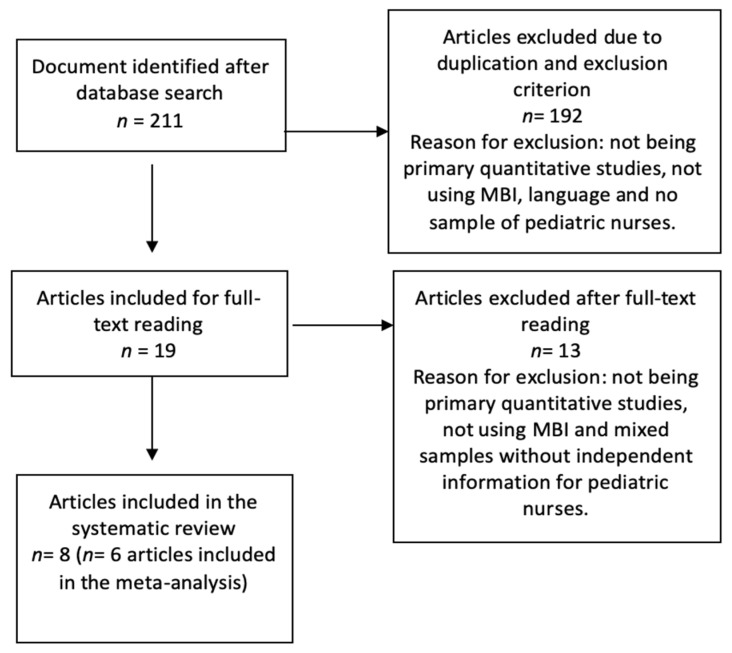
Flow diagram of the publication search process.

The initial literature search produced 211 articles, of which 192 were eliminated according to the inclusion and exclusion criteria. After reading the full text and the forwards and backwards search, the final study sample was composed of eight articles with six studies including the necessary information for the meta-analysis (see Figure 1). Three outcome categories were then defined: “burnout prevalence and meta-analytical estimation”, “burnout levels” and “burnout risk factors”.

### 4.1. Burnout Prevalence and Meta-Analytical Estimation

Two studies reported a high prevalence of EE, with values of 48.4% [34] and 57.14% [35], while another three obtained values ranging from 24.6 to 34.1% [36,37,38]. The lowest prevalence, 15.6%, was obtained by Italia et al. [39]. 

The highest prevalence of D (29.8%) was reported by Zanatta and Lucca [38]. The values reported by other researchers ranged from 8.6 to 25% [34,35,36,40], with the exception of Gallagher and Gormley [37], who measured only 3.3% of high D.

Finally, a low prevalence of PA (which is measured in the inverse sense to that of the other dimensions) under 20% was only found in two studies [37,41], which reported values of 13.3% and 16.7%, respectively, while the remaining studies obtained higher values, ranging from 22.8 to 41% [34,35,36,38]. The above findings are presented in detail in Table 1. 

Egger’s test results showed no publication bias (*p* > 0.05 for all dimensions). In the sensitivity analysis, the prevalence values remained unaltered when each study was eliminated in turn from the meta-analysis.

Cochran’s Q values obtained (for EE, D and PA, respectively) were 16.17 (*p* < 0.05), 27.85 (*p* < 0.05) and 18.85 (*p* < 0.05). The I^2^ index value was 75.3% for EE, 85.6% for D and 73.5% for PA, reflecting the presence of heterogeneity among the three dimensions of burnout.

**Figure 2:**
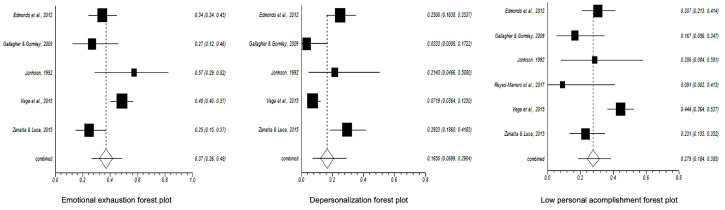
Forest plots of (**a**) emotional exhaustion, (**b**) high depersonalization and (**c**) low personal accomplishment.

The meta-analytic estimation of the prevalence of high EE, high D and low PA were, respectively, 37% (95% CI = 26–48%), 16% (95% CI = 6–29%) and 27% (95% CI = 18–38%) with a sample of *n* = 361. The corresponding forest plots are shown in Figure 2.

### 4.2. Levels of Burnout

Only two of the studies [37,38] presented the prevalence detailed by each of the dimension, according to the burnout scores obtained (Table 2). According to these studies, 24.6% and 26.7%, respectively, of the sample population experienced high levels of EE, while 46.7% and 49.1% experienced moderate levels and 26.3 and 26.7% low levels. The values obtained, thus, were very similar in each case. These researchers obtained high levels of D in 3.3% and 29.8% of the respective samples, while moderate D was observed in 33.3% and 43.9%, and low scores were obtained for the remaining 26.3% and 63.3%, respectively. Low levels of PA were obtained for 16.7% and 22.8% of the respective samples, moderate ones for 33.3% and 52.6%, and high ones for 24.6% and 50%.

### 4.3. Risk Factors for Burnout in Paediatric Oncology Nurses

The data for sociodemographic risk factors are shown in Table 3. In this respect, some studies reported higher levels of burnout among female nurses [40,41], while others showed a statistically significant relationship between married status, male gender and increased EE [37,38]. In other studies, childless status was found to be related to increased D among younger nurses [34]. Edmonds et al. [36] observed that young nurses presented higher levels of EE than older ones; on the other hand, burnout has also been associated with being 40 years or more [34,39,41].

In relation to organisational and other occupational factors, Reyes-Marrero et al. [40] reported an association between burnout and a relative lack of professional experience, while Liakopoulou et al. [40] found this same factor to be associated with increased D. For Gallagher and Gormley [37], the three dimensions of burnout were related to inexperience, i.e., the lower the level of experience, the greater the EE and D and the lower PA. 

Johnson [35] found that the lack of peer support, together with frustration and overwork, significantly increased EE. Similarly, Vega et al. [34] showed that the lack of social support and team cohesion were related factors, while Liakopoulou et al. [40] showed that EE was also higher when the nurses´ professional role was inadequately defined. In one study, the complexity of patients’ conditions was found to be related to increased EE [37], while other authors concluded that working in night shifts increased the possibility of suffering burnout [37,41]. As a protective factor, the presence of adequate support systems was found to enhance feelings of PA [37]. 

Finally, with respect to psychological factors, a statistically significant relationship has been observed between nurses’ witnessing suffering and death and their experience of greater EE [36,38].

## 5. Discussion

Regarding the dimensions of burnout, our literature search also revealed a study of neonatal intensive care nurses, which reported findings similar to those obtained in the papers selected for detailed analysis, with high levels of EE and moderate levels of D [42].

Another study with oncology nurses measured a moderate degree of EE, high levels of D and low PA. These nurses were subjected to a heavy workload and sometimes to conflicts with family members. Moreover, they were frequently confronted with the death of their patients. These factors, among others, explain why burnout is accentuated in this type of hospital unit, especially when the patients involved are children [43]. In another study, conducted in adult and paediatric oncology units [44], the level of nursing experience was not found to be associated with EE, which contrasts with the findings of Gallagher and Gormley [37] in this respect. According to Neumann et al. [44], nurses caring for paediatric patients presented lower scores for EE than those responsible for adult oncology patients.

The following conclusions were drawn regarding the association between sociodemographic factors and burnout: Female nurses showed a higher prevalence of burnout compared with males [45]. Moreover, most female nurses combine occupational duties with domestic work, which tends to increase their feelings of D [46]. In addition, married nurses have higher levels of EE, which may be because of having to cope with the stress of work and with family tensions, which can damage their self-esteem; consequently, marital status is a potential risk factor for the development of burnout [23]. The possible association between having children and suffering burnout is a question of some controversy. In general, however, parenthood is assumed to be a protective factor against burnout [47]. Some research findings have related burnout directly to greater youth and to lack of experience. If so, age would be a protective factor, as older nurses would have more experience, being better equipped to deal with problems arising in the workplace [14,48].

Regarding organisational and occupational factors, Ferri et al. [49] studied the relationship between burnout and professional experience, contrasting nursing students with more experienced nurses. The students presented lower levels of burnout, presumably because they were initially highly motivated and involved, and had strong feelings of empathy towards their patients. However, other studies have shown that older nurses are better at developing coping strategies due to their more extensive work experience [14,50,51].

It has also been suggested that the working environment may be related to PA. Thus, in favourable working conditions, nurses would perform their duties to an optimal degree, and so their PA would be enhanced [22]. On the other hand, overload (in terms of length of the working day or lack of time for patient care) contributes to EE [14,26,52]. Particular complexity of patients’ medical conditions and requiring additional nursing time and attention are other risk factors for burnout [53]. These negative aspects of the working environment can provoke frustration and dissatisfaction, as nurses find themselves unable to meet expectations [54,55]. Other consequences include poor working relations with colleagues, a lack of cohesion within teams and inadequate social and occupational support [14]. Indeed, support from supervisors is essential to nurses’ job satisfaction, reducing absenteeism and staff turnover [54,56]. Another important aspect is that nurses’ roles and functions should be clearly defined; if they are not, this produces added stress and can provoke dissatisfaction and problems within the medical team, aggravating EE [57]. These consequences could be minimised by the deployment of sufficient nursing staff and by the equitable distribution of responsibilities [58].

Rotating shift work seems to provoke higher levels of burnout, as it produces changes in circadian rhythms and therefore decreases the quality of sleep, resulting in insomnia, fatigue, anxiety, loss of concentration and behavioural changes, which are prejudicial to the provided care [59,60,61]. Working night shifts, in particular, has been related to increased burnout, since biological rhythms are altered, causing sleep and/or appetite disorders, daytime tiredness and alterations to personal life [60,62].

Finally, psychological factors must be taken into account, as emotional suffering is inherent to oncology healthcare, a unit in which death is constantly present and must be coped with [26,63]. In addition, nurses with high levels of EE may suffer from anxiety and even depression [64,65]. Furthermore, other psychological alterations related to burnout such as sleep disorders or behaviour changes that can occur due to overwork, insufficient staff or workday prolongation should be taken into account when addressing the problem of burnout [54,66].

Other burnout-related psychological alterations include sleep disorders, stress and behavioural changes resulting from overwork, insufficient staff or prolonged working hours, and should be taken into account in order to prevent or remedy the occurrence of the syndrome [54,66,67]. Accordingly, programmes should be developed and applied to address the work-related and psychological risk factors identified above [17,26,63,64].

### 5.1. Implications for Healthcare Practice

Burnout prevention would avoid a decrease in the quality of nursing care and more specifically in paediatric oncology nurses, as patients in this service have very specific characteristics [68]. For this, nurse managers must promote an adequate working environment, which can be achieved by reducing the overload and providing social support at work so that relationships with colleagues and patients improve [69]. The result will be nurses more satisfied in their work and with a better ability to cope with problems [70].

One way to prevent burnout and promote a suitable working environment would be implementing intervention programs focused on nurses. In this respect, some papers have described the application of group-focused interventions based on relaxation and mindfulness techniques [71,72,73,74], or on enhanced communication [75].

### 5.2. Study Limitations

The present study has certain limitations. The number of papers included in the review was very limited due to the scarcity of original sources providing information about burnout among paediatric oncology nurses. Moreover, in some studies, the sample was mixed and it was not possible to obtain independent burnout data for paediatric oncology nurses. In addition, both the level of evidence and the degree of recommendation are low since the analysed studies are descriptive. Finally, the articles considered differ in the date and language of publication, which may also have led to some disparity in the results presented.

## 6. Conclusions

Analysis of the literature shows that paediatric oncology nurses are especially vulnerable to EE. In addition, they present a moderate level of D, but in most cases, the level of PA remains high. The main risk factors reported are age, gender, marital status, shift work and experience. On the contrary, the presence of a good working environment and social support are protective factors. Appropriate interventions by supervisors can enhance the working environment and help prevent burnout and/or reduce its impact.

Finally, the fact that very few research papers on this subject have appeared underlines the fact that further investigation is needed to identify strategies that can prevent burnout, and thus improve nurses’ quality of life and the quality of care provided to patients.

## Figures and Tables

**Table 1:** Prevalence of high emotional exhaustion (EE), high depersonalisation (D) and low personal accomplishment (PA).

Author, Year. Country	Sample *n*	% of High EE	% of High D	% of Low PA
[36]	88	34.1%	25%	30.7%
[37]	30	26.7%	3.3%	16.7%
[35]	14	57.1%	21.4%	28.5%
[41]	11	-	-	13.3%
[34]	153	48.8%	16%	41%
[38]	65	24.6%	29.8%	22.8%

**Table 2:** Distribution of the three Maslach burnout inventory (MBI) components and their respective percentages.

Author, Year	EE (%)			D (%)			PA (%)		
	High	Medium	Low	High	Medium	Low	High	Medium	Low
[37]	26.7	46.7	26.7	3.3	33.3	63.3	50	33.3	16.7
[38]	24.6	49.1	26.3	29.8	43.9	26.3	24.6	52.6	22.8

**Table 3:** Characteristics of included studies (*n* = 8).

Author, Year, Country	Study Type	OCEBM	*n*	Version of MBI	Mean EE	Mean D	Mean PA	Risk Factors
[36]	Descriptive, cross-sectional	LE: 2C GR: B	88	MBI	22.3	6.1	35.9	- Younger nurses reported significantly higher rates of EE. - Work experience affects the burnout levels. Nurses with less experience present higher levels of EE. - Psychological morbidity correlates with the EE.
[37]	Exploratory, descriptive	LE: 2C GR: B	30	MBI-HSS	26.7	3.3	16.7	- Novice nurses have higher levels of EE and D and lower levels of PA. - The complexity and critical nature reported high levels of EE. - Support systems are associated positively with PA. - Shift work, in particular night shift work, is associated with high levels of EE and D.
[39]	Pilot study	LE: 2C GR: B	16	MBI-HSS	ND	ND	ND	- Older nurses experience high levels of burnout.
[35]	Descriptive, correlational	LE: 2C GR: B	14	MBI-HSS	26.4	8.2	34.7	- Overwork correlate positively with EE more than D. - Demanding role with stressed parents tends to score higher in EE. - Lack of companionship and co-worker gossip are correlated with higher level of EE than D. - Levels of frustration are related to EE levels more so than D levels.
[40]	Comparative and descriptive, correlational	LE: 2C GR: B	37	MBI-HSS	27.5 ± 9.5	5.2 ± 4.9	37.8 ± 5.8	- D is related to not having children. - D increases in nurses with less experience. - Decreased role clarity is positively correlated with EE and negatively with PA.
[41]	Descriptive, cross-sectional	LE: 2C GR: B	11	MBI	SD	SD	SD	- The female gender is associated with higher levels of burnout. - Burnout levels are associated with the nurses’ age. Nurses older than 40 years have an average level of burnout. - Nurses on the night shift have higher levels of burnout. - Burnout levels are related to less work experience.
[34]	Descriptive, cross-sectional	LE: 2C GR: B	153	MBI	48.4	16	41	- Burnout among females is significantly higher. - Older nurses (more than 40 years old) present higher levels of burnout. - Demographic variables such as marital status, age, years of experience and number of children are not related to burnout prevalence.
[38]	Descriptive, cross-sectional	LE: 2C GR: B	57	MBI	24.6	29.8	22.8	- Marital status is related to EE. Married nurses are more prone to EE. - Health problems are related to high levels of EE.

Abbreviations: EE: emotional exhaustion; D: depersonalisation; PA: personal accomplishment; LE: level of evidence; GR: grade of recommendation; OCEBM: levels of evidence of the Oxford Centre for Evidence-Based Medicine; MBI: Maslach burnout inventory; MBI-HSS: Maslach Burnout Inventory-Human Services Survey; ND: no data.
